# Wild Bee Species (Hymenoptera: Apoidea: Anthophila) of Three Western Provinces of Cuba: A Century of Temporal Dynamics

**DOI:** 10.1007/s13744-025-01282-6

**Published:** 2025-06-18

**Authors:** Sandra Duarte, Lise Ropars, Nathalie Machon, Laure Desutter-Grandcolas

**Affiliations:** 1 CESCO, Muséum National d´Histoire Naturelle, Paris, France; 2 ISYEB, Muséum National d´Histoire Naturelle, Paris, France; 3 Museo Nacional de Historia Natural de Cuba, La Habana, Cuba

**Keywords:** Urban biodiversity, Ecology, Pollinator, Conservation, Species richness, Museal collections

## Abstract

Bees, which play a vital role in the reproduction of plant, are in decline due to multiple factors. In tropical islands, despite their growing extent, urban areas are less explored and sampled compared to natural areas. Historical collections and online databases can then provide information on the temporal dynamics of pollinators as well as ecological information, providing a proxy for deficient sampling. This study investigates the diversity and ecological characteristics of the wild bee community and its temporal dynamics in the Havana province of Cuba. Cuba is a biodiversity hotspot in the Caribbean and hosts the highest bee biodiversity in the Antilles. Here, we analyzed 1,322 records (of which 1,067 new) from various sources (online databases, literature, and historical natural collections during the XIX Century). In the studied area our data attest the presence of 52 species (23 genera, 4 families), of which 47 are native (14 endemics) and four are introduced species Three species categorized as "critically endangered" for the country were recorded; 41 species are polylectic (78.8%) and 11 kleptoparasitic (21.2%). Most species were found during the rainy season (49 species vs 39 species recorded in dry season). Regarding the variation of the wild bee population over the years, one third of the recorded species have not been observed since the last century, while about 70% of the species persist. Analysis of a century data underscores the importance of conserving the wild bee community in these provinces.

**Supplementary Information:**

The online version contains supplementary material available at 10.1007/s13744-025-01282-6.

## Introduction

**Fig. 1:**
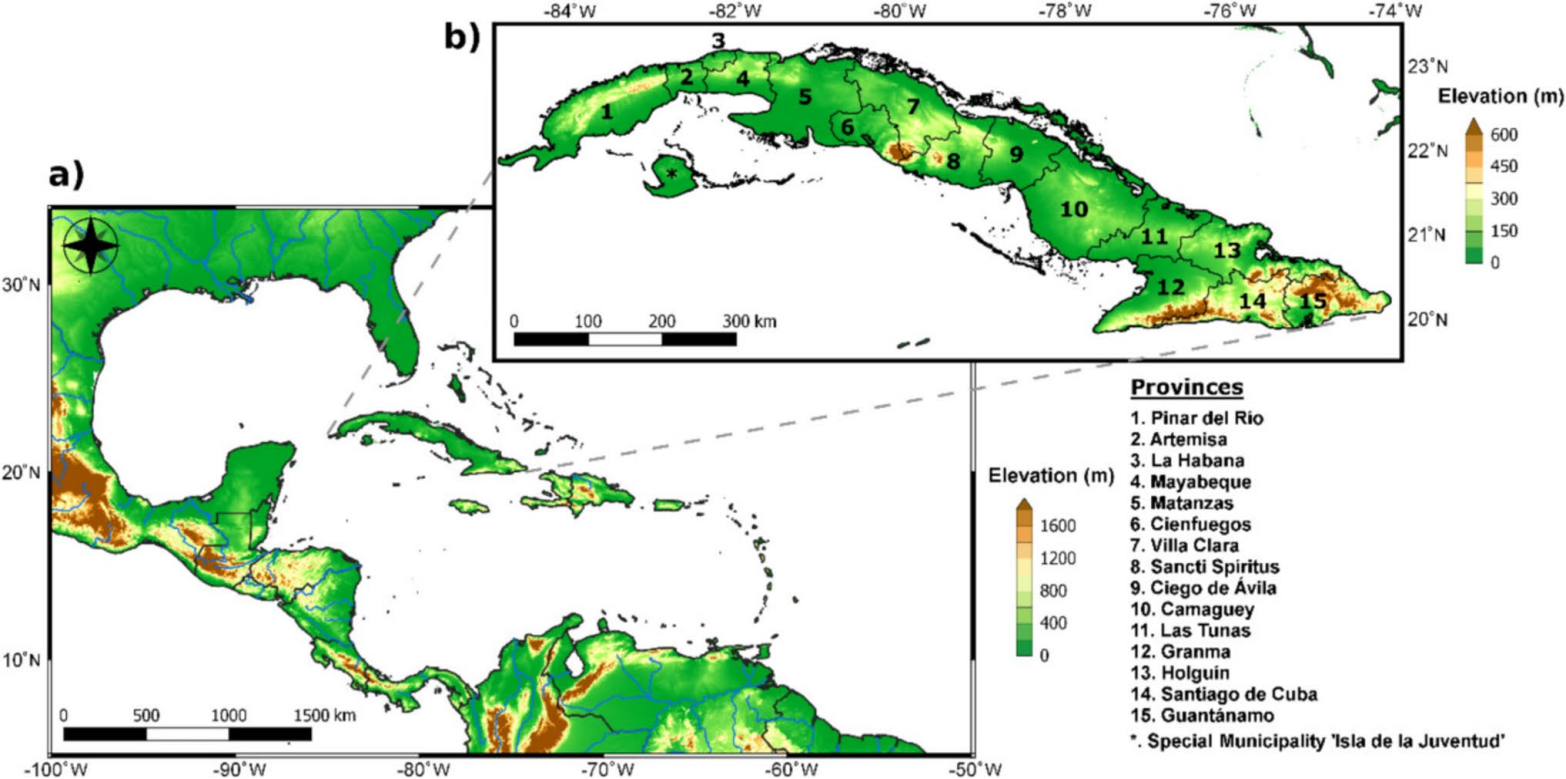
Geographic location of Cuba in the Caribbean region (**a**) and provinces of Cuba (**b**). Taken from Fernández–Alvarez et al. 2020

With approximately 21,000 described species (Ascher & Pickering. 2020), bees (Anthophila) are the most important diurnal pollinators of angiosperms (Michener 2000, 2007) and play a crucial ecological role in urban and natural ecosystems (Genaro 2007; Oliveira & Schlindwein 2009; Egerer et al. 2019). The Caribbean is considered a biodiversity hotspot (Myers et al. 2000) and possesses a high level of biotic endemism (Genaro 2001a; Genaro 2006). As the largest island in the Caribbean, with a land area of 110,860 km^2^, Cuba encompasses a multitude of habitats and ecoregions that currently host the highest biodiversity of bees in the Antilles (Fig. 1) (Genaro 2007).

The Cuban bee fauna is one of the richest in the insular Caribbean, with 95 described species (Genaro 2008), documented through a rich, although scattered, literature. Most of these species have been collected in preserved or mountainous natural areas with high levels of endemism (Genaro 2008; Duarte and López 2019). Previous species lists have been published for different regions of Cuba, i.e. the Isla de la Juventud (34 spp) (Genaro 2004), the Jibacoa-Cayajabos area (García et al. 1973), the Granma province (27 spp) (Fernández Triana et al. 2002), the mountainous massifs of the Eastern region (56 spp) (Portuondo and Fernández-Triana 2004), and two areas of Pinar del Río (58 and 42 spp respectively) (Breto 2021a, b). Some punctual inventories have also been carried out in several protected areas (Fong et al. 2005a, b; Maceira et al. 2005, Maceira et al. 2006). According to Fernández Triana (2005), the Eastern region has been the most inventoried area in Cuba, with 14 published inventories, while the Occidental region is documented through three published inventories (Genaro 2004; Breto 2021a, b).

No published data exist for Havana and the surrounding provinces. In this case, unstructured and opportunistic historical collections of specimens may reveal a rich source of information to evaluate the diversity of taxonomic groups (bees) in the area (Bartomeus et al. 2013; Zimmermann et al. 2023). Among these taxonomic groups, the genus *Lasioglossum* Curtis presents a particularly complex subgeneric classification, and even recent phylogenetic and molecular studies have not comprehensively covered the Caribbean species groups (Gibbs et al. 2013; Gibbs 2018). Analysis of historical collections has been acknowledged as the best way to study wild bee diversity in unsampled areas (Mathiasson and Rehan 2019; Meunier et al. 2023). Specimens preserved in museums may also provide ecological information such as interactions, morphological traits or diet, based on the data collected through time. Bartomeus et al. (2013) and Zimmermann et al. (2023) demonstrate the feasibility and the power of the study of historical collections to identify long-term changes in biodiversity, species abundance, morphology, and pollination services. Online databases are equally useful and complementary, as shown by Zattara and Aizen (2021), who described richness patterns based on Global Biodiversity Information Facility (GBIF) records.

The objective of this study is to evaluate the diversity of wild bees in the Cuban provinces of Havana, Artemisa and Mayabeque, and to describe their ecological and temporal characteristics, using historical records, online databases and the literature. We will answer the following questions: (i) what is the diversity of wild bees in these areas? (ii) what are their ecological characteristics? (iii) can we put in evidence patterns of temporal trends of richness, dietary habits and phenology in bee communities between 1900 and 2024?

This work is the first list of bee species in the studied provinces. It will constitute a baseline for future studies of species richness, distribution, food habits and phenology of bee species communities through time and according to landscape changes.

## Material and Methods

### Data Collection

**Fig. 2:**
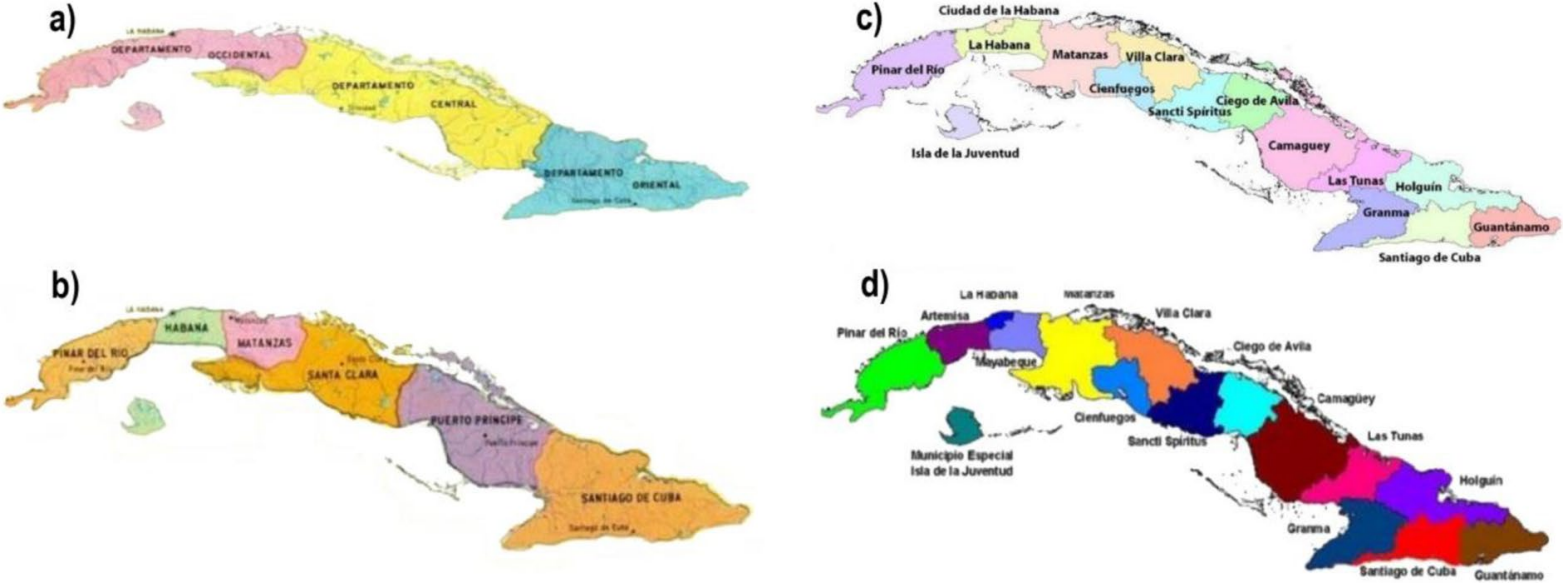
Administrative divisions of Cuba through the years 1827 (**a**), 1878 (**b**), 1976 (**c**) and 2011 (**d**). Taken from Méndez and Lloret 2021. Labels of each map from left to right: **a**) Departamento occidental, Departamento central, Departamento oriental; **b**) Pinar del Rio, Habana, Matanzas, Santa Clara, Puerto Príncipe and Santiago de Cuba; **c**) Pinar del Rio, Ciudad de la Habana, Habana, Isla de la Juventud, Matanzas, Villa Clara, Cienfuegos, Sancti Spíritus, Ciego de Ávila, Camagüey, Las Tunas, Holguín, Granma, Santiago de Cuba and Guantánamo and **d**) Pinar del Rio,, Artemisa, La Habana, Mayabeque, Isla de la Juventud, Matanzas, Villa Clara, Cienfuegos, Sancti Spíritus, Ciego de Ávila, Camagüey, Las Tunas, Holguín, Granma, Santiago de Cuba and Guantánamo

Data were gathered from bibliographic sources, online databases, from several Museums collections and from collections made by the first author in urban farms in Havana between 2018–2019 and 2023–2024 (Appendix 1). All the specimens collected in"Havana","La Habana","Havane","Artemisa"or"Mayabeque", were included. The label"Havana"actually reflects a political and administrative division of Cuba, which delimitation has changed through time (Fig. 2). In 1827, Cuba was divided into three provinces, e.g. the Occidental (including the capital La Havana), Central and Oriental provinces. From 1878 to 1976, Cuba was divided into six provinces, one of which, i.e. La Havana, included the city of La Havana, its surroundings and the Isla de la Juventud. In 1976, this province was split again into two administrative units, both with Havana as their capital. Finally, in 2011, the administrative organization of Cuba was reconsidered and three provinces were defined in the"old"Havana province, e.g., Havana, Artemisa, and Mayabeque (Fig. 2) (Méndez Delgado et al. 2021). Collection dates for specimens from"Havana"range from 1867 to 1999 and may encompass localities from these three recently defined provinces.

The following Museum collections ordered alphabetically have been considered: in Cuba: Instituto de Ecología y Sistemática (IES) and Museo Nacional de Historia Natural de Cuba (MNHNCu) (Havana), in France: Muséum national d´Histoire naturelle (MNHN) (Paris) and in United Stated of America: American Museum of Natural History (AMNH) (New York), Kansas University Natural History Museum (KU-NHM) (Kansas), Smithsonian Institution National Museum of Natural History (SI-NMNH) (Washington).

All distribution data inside the Neotropical region were completed/corroborated with online databases: Global Biodiversity Information facility (GBIF) (www.gbif.org), Catalogue of life (CoL) (www.catalogueoflife.org) and Moure Catalog for Neotropical bee species (Moure bees) (moure.cria.org.br) Some of these online databases, as GBIF, have access to data from other websites such as iNaturalist and for species occurrence data.

For each apoid specimen, all available label data were recorded, including location, date, collector and sex. Species name was noted if available, and corrected if necessary; unidentified specimens have been identified by the first author. For species distributions, biogeographic region and subregion follow Morrone (2014), and the countries of species occurrence are provided.

The data from the MNHNCu databases, mostly produced by J. Genaro (MNHNCu), were examined by the first author. The specimens deposited in the MNHN were identified/reidentified by the first author, and for some genera by several colleagues according to their expertise. Photos were sent to Jason Gibbs (University of Manitoba), who kindly assisted with identification of *Lasioglossum* specimens. Michael Engels (AMNH) helped in the identification of the family Halictidae. All MNHN specimens were attributed unique inventory numbers.

The taxonomy of Apoidea has greatly changed over the last 150 years (Meunier et al. 2023), and current taxonomic names have been applied. Bee nomenclature follows Michener (2007) updated in light of recent taxonomic advances, such as genus definitions. To verify the validity of names different databases were checked. Additionally, the latest taxonomic works were reviewed, including Carman and Packer (1996), Genaro (1998, 2001a, b, c, 2004, 2008, 2016), Genaro and Franz (2008), Rightmyer (2006, 2008), Gibbs (2011, 2018), Engel et al. (2012), Vivallo (2014), Correia da Rocha and Packer (2017), Sheffield et al. (2020, Sheffield et al. 2021), Genaro and Breto (2022), and Moure et al. 2022a, b).

A list of the wild bees of Havana, Artemisa and Mayabeque is presented alphabetically organized by family, genus and species, with the ecological characteristics of each species (Appendix 2). The resulting databases are shared as Online resource1 in Darwin Core format (GBIF, 2023).

### Conservation Status and Species Traits

The species conservation status and frequencies (common or rare) was assigned based on the Red List of Cuban Species (Hidalgo-Gato González et al. 2016).

Several ecological characteristics were described, including feeding strategy (polylectic, kleptoparasitic) taking into account published diet studies, distribution (endemic species, shared in the region, introduced in Cuba), according to original descriptions and communication with specialists (Engel M, Genaro J, and Gibbs J). According to the geographic location of Cuba, the climate is characterized by two main hydroclimatic periods: winter or “dry season” (November to April) and summer or “rainy season” (May to October) (Fernández-Alvarez et al. 2020). Based on this dichotomy, the records were grouped to detect which months were the most collected, and which species were collected each month.

For the seasonal evaluation, we used all records documenting the month, which corresponds to 1237 records belonging to 52 bee species. To test the existence of significant differences between the two seasons (dry and rainy), a student's t-test was performed on R.

### Data Analysis

All data analyses were conducted under R software version 4.2.0 (R Core Team 2020), using the packages sf, FactoMineR, factoextra, vegan and ggplot2 (Wickham 2016).

#### Time Periods

The historical collection records spanned from 1867 to 2024 (1202 records). Due to gaps in species collections between years, they were grouped into four periods, each 25-years in duration and including a minimum of five species. Records from 1867 to 1889 were excluded because of lack of sufficient data. The resultant time-periods were: 1900–1925, 1926–1950, 1951–1975, 1976–2000 and 2001–2024.

To assess which species have persisted or declined over the 125 years of data we have, we analyzed the presence of the 52 wild bee species through each of the 25-year periods. For the analysis of the most recent 25-year period, 2001–2025, we took into account the sampling conducted by the first author in Havana and the observation data from iNaturalist. From iNaturalist, only validated data were collected for all the provinces that compose the western region (Pinar del Río, Artemisa, Havana, Mayabeque and Matanzas).

To determine the efficacy of bee community sampling events, we combined all bee captures across years and explored species richness using an accumulation curve and estimating asymptotic species richness. Using the vegan package (Oksanen et al. 2018) for R Core Team 2020 we generated a species accumulation curve. The expected total species richness was assessed using the Chao and Jackknife estimator due to the characteristics of the data (many singletons) (Fortel et al. 2014). Dominant (> 50 observations) and rare (< 8 observations) species were determined within the bee population.

#### Distribution in Neotropical Region and in Western Provinces

To assign biogeographic regions based on distribution, we adopted the nomenclature of the Neotropical Region proposed by Morrone (2014), who considered two subregions (Antillean and Brazilian). South Florida was incorporated into the Bahamas province.

1018 records had detailed locality information (48 spp, 117 localities). The geographic information from labels was compiled, and locality data were verified and updated according to the new geographical distribution of Cuba (Méndez Delgado et al. 2021). Although these records span over a century, they are considered “new” records, as they have not been published before. To complement this information, geographical coordinates, absent in the labels, were added when possible. Selecting the most precise locality as possible, GPS coordinates were taken for a point based on the information provided by the various sources, including Google Earth (earth.google.com) and Coordonnées GPS (www.coordonnees-gps.fr).

## Results

### Diversity of Wild Bees

#### Species Composition

Analyzing various sources that mentioned specimens labelled from Havana (Online resource1), we gathered 1,322 records, of which 1,067 are new records for the regions that had not been published. The highest number of individuals is in the collections of MNHNCu (46.1%), followed by IES (22.8%) and MNHN (16.9%). The KU-NHM collection represents 7% of the recorded individuals, and this data has been already published (https://www.amnh.org/research/invertebrate-zoology/collections/hymenoptera-apoidea). The other collections accounted for 7.2% of the recorded data.

In the three provinces, 52 species (52.6% of the Cuban wild bees described) of wild bees were recorded out of the 95 species documented for Cuba (Appendix 2, Online resource1). They belong to 23 genera (31 for Cuba) and all the 4 families present in the country. The number of species found in each historical collection was as follows: 46 species in MNHNCu, 27 species in IES, 26 species in both museums MNHN and KU-NHM, 6 species in AMNH, 3 species in USNM, 2 species in INHS, 2 species in MZC and 2 species in SI-NMNH, and 1 species in each place of CUIC, FSCA, LACM, NMNH, PCYU, USNM (Appendix 1, Online resource1).

#### Conservation Status of Wild Bee Species

Thirty-three species were common, i.e. with more than eight reports and seven species with more than 60 individuals: *Lasioglossum parvum*, *Halictus. poeyi*, *Agapostemon femoralis*, *Agapostemon poeyi*, *Apis mellifera*, *Nomada cubensis*, and *Ceratina cyaniventris*.

Some species were uncommonly recorded in these provinces. Only 16 species listed show less of eight reports i.e. *Augochlora magnifica*, *Augochlora praeclara*, *Centris fasciata*, *Ceratina cockerelli*, *Coelioxys sannicolarensis*, *Coelioxys tridentatus*, *Colletes submarginatus*, *Epeolus pulchellus*, *Lasioglossum normalis*, *Megachile. apora, Meg. concinna, Meg. curta*, *Mesoplia cubensis*, *Triepeolus roni* and *T. wilsoni* and *Xeromelecta alayoi*. Three species are classified as"Critically Endangered"on the Red List of Cuba: *Coe*. *sannicolarensis*, *Coe*. *tridentatus* and *Hy*. *limbifrons*.

### Ecological Characteristics of Wild Bees

#### Origin and Distribution

The bee fauna found in this study consisted mostly of native species (90.4%, 47 species), with a smaller proportion of introduced species (9.6%, 5 species). The introduced species included *A. mellifera*, *Mel. beecheii*, *Meg. concinna, Meg. lanata and Meg. rufipennis*. Out of the total native species, 13 species (25%) were biogeographically defined as endemic (Appendix 2; Online resource1). These endemic species belong to the genera *Anthophora* (1), *Ceratina* (1), *Epeolus* (1), *Melissodes* (3), *Mesoplia* (1), *Nomada* (2), *Hylaeus* (1), *Agapostemon* (1), *Lasioglossum* (1) and *Coelioxys* (1) (Appendix 2, Online resource1). In addition to these Cuban endemics, 25 species are restricted to the Lesser Antilles (49%) and occur on various islands.

#### Distribution of Sampling in Western Cuba

**Fig. 3:**
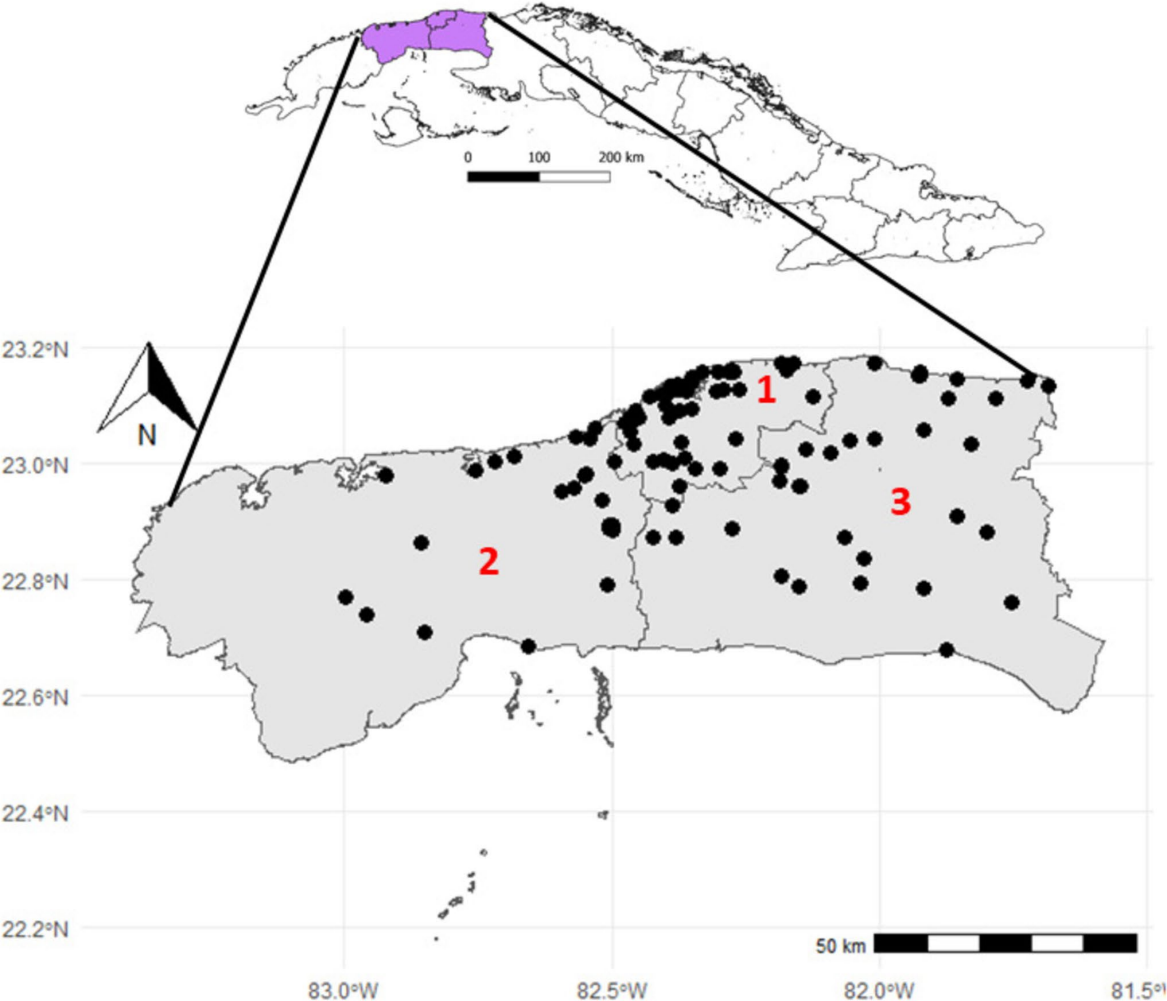
Distribution of wild bees in the three western Cuban provinces (occurrence map). Provinces: Havana (1), Artemisa (2), and Mayabeque (3)

Concerning collection localities of the 1018 records with complete collection locality data, 117 sampling localities were identified for 48 species of wild bees (4 species without complete locality data) in Havana, Mayabeque and Artemisa (Online resource1). In the province of Havana 51 localities were sampled, while Mayabeque comprises 38 localities, according to the current political-administrative division, and Artemisa was the one that sampled the least, with a total of 28 localities (Fig. 3).

**Fig. 4:**
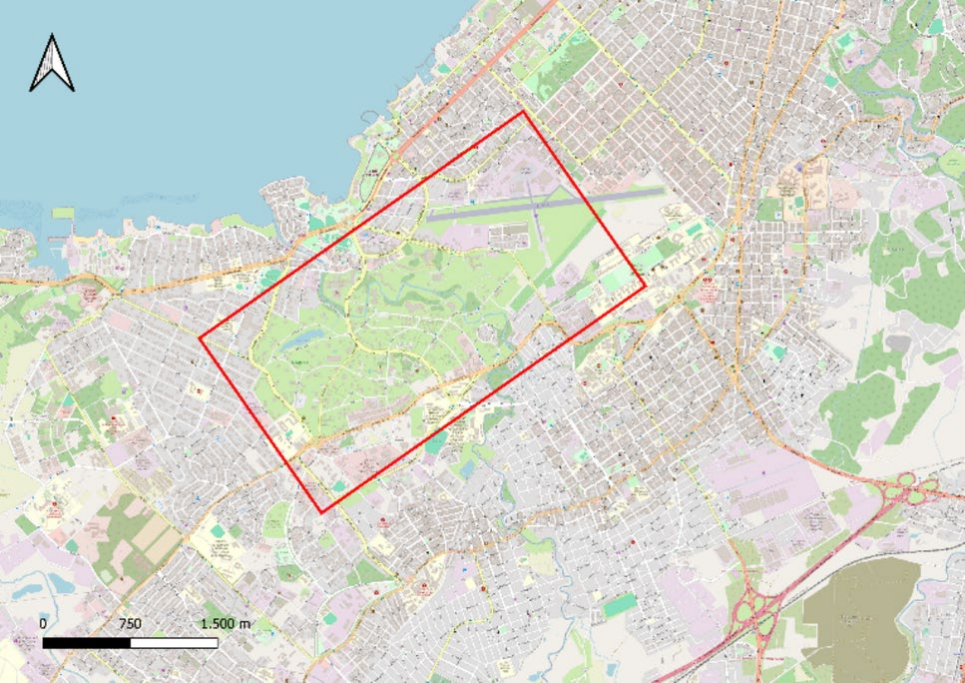
Densely sampled site in Havana: El Laguito, Havana

The highest sampling frequency was observed in Santiago de Las Vegas in Boyeros and El Laguito in Playa (Havana), with 157 records encompassing 35 species. In Artemisa, El Mariel accounted for 99 out of 804 records and five species, whereas in Mayabeque, sampling was concentrated in Güines, yielding 64 records each for seven species (Fig. 4).

#### Feeding Habits of Species and Seasonal Distribution of Sampling

The feeding habits of the species varied between polylectic non parasitic (80.8%, 42 spp.) and kleptoparasitic (19.2%, 11 spp) (See Appendix 2, Online resource1). The 11 kleptoparasitic species belong to the genera *Epeolus pulchellus*, *N. cubensis*, *N. pilipes*, *N. cruralis*, *T. vicinus*, *T. roni*, *T. wilsoni*, *Coe. rufipes*, *Coe*. *sannicolarensis*, *Coe*. *tridentatus* and *X. alayoi*.

**Fig. 5:**
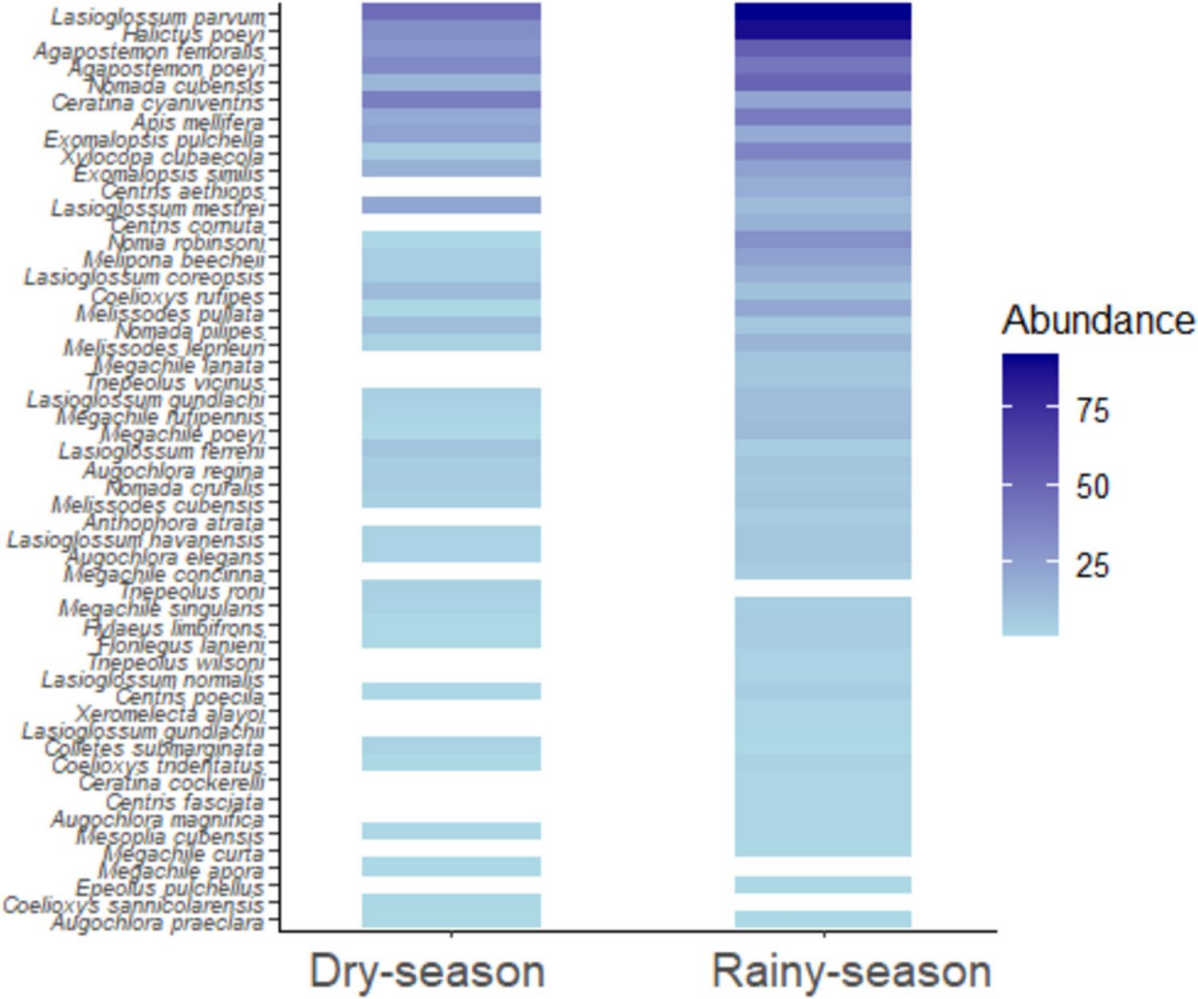
Seasonal distribution of wild bee species occurrence. The color intensity gradient represents the distribution of catches for each species over the study period

When assessing the seasonal distribution of sampling, no significant differences (p-value = 0.9) were observed in the abundance of species per period (Fig. 5; Appendix 2). An analysis within months by season showed that May registered the highest number of records (156), during the rainy season, whereas September recorded the lowest (42). In the dry season, April recorded the highest number of collects (207), while November exhibited the lowest (18). The most collected species in each of these months were: *L*. *parvum* in April (41 ind), *H*. *poeyi* in May (21 ind) and *Me*. *cubensis* (4 ind) in November.

The number of species per season was slightly higher in the rainy season (49) than in the dry season (39). Sixteen species of wild bees were present in only one season and 32 species in both seasons. The most abundant species were *L. parvum* (155 ind.), *H. poeyi* (121 ind.), and *A. femoralis* (81 ind.) in both periods. Six species were observed only in dry seasons and 10 only in rainy seasons.

### Temporal Variation of Diversity, Dietary Habits and Phenology in Wild Bee Species Over 125 Years

#### Diversity Variation of Wild Bees. Persistence and Decline of Wild Bees Over Time

The oldest records trace back to the year 1867, featuring nine collected specimens belonging to three species (*A. femoralis*, *A. elegans*, and *Ex. pulchella*). The most recent species records for Havana are from 2024 including the species *A. mellifera*, *C. cyaniventris*, *Ex. pulchella*, *H*. *poeyi*, *M. concinna*, *Mel. beechei* and *X. cubaecola*. All data for 2024 were collected on urban farms as part of a project conducted by the first author.

Nineteen species were present in more than three periods. Eleven species were present in only one period, and seven species were present in all five periods: (*A*. *femoralis*, *A*. *poeyi*, *A*. *mellifera*, *C*. *cyaniventris*, *Ex. pulchella*, *H*. *poeyi* and *N*. *cubensis*). Of the species present in a single 25-year period, the 1976–2000 period was the most outstanding.

Throughout 125 years of data from different sources, the dominant (7), intermediate (25) and rare (20) species were determined according to the frequency of observation. The three dominant species were: *L*. *parvum,* H. *poeyi*, *A*. *femoralis* followed by *A*. *poeyi*, *A. mellifera*, *N. cubensis*, and *C. cyaniventris*. Among the rare species, 16 species had a frequency of observation lower than five times and four species were recorded only once (*Coe*. *sannicolarensis* (unknown), *Ex*. *pulchellus* (1975–2000), and *Meg*. *apora* (1975–2000).

When analyzing how the species dominance varied over the 125 years, it was observed that before 1900, *A*. *femoralis* (eight ind.) was the most collected bee, a species that appears among the most dominant in the wild bee population. In the 1900–1925 period, the most commonly collected species from the western region in zoological collections were *A*. *mellifera* and *N*. *cubensis*, followed modestly by *C*. *aethiops*. In the 25-year period that followed, the abundance of specimens declined, with *Meg*. *rufipennis* and *A*. *poeyi* standing out. In the 1951–1975 period, there was an explosion of L. parvum in the collections, followed closely behind by *A*. *poeyi*. In the following 25-year period, when more individuals were found, *H*. *poeyi* and *L*. *mestrei* were the most abundant species. In the most recent period, 2001–2025 the small bee, *A*. *mellifera* was the most collected, followed by *C*. *cyaniventris*.

In the analysis of the 52 species across the 125 years of data, it was observed that 31.5% of the species have not been observed since the last century, some for more than 100 years, such as *C*. *cornuta* (1909) and *Aug*. *magnifica* (1911). However, 68.1% of the species have been observed in recent years, even 7 species not previously collected were reported in this period in one of the provinces (Pinar del Río): *Caupolicana nigrescens*, *C*. *tarsata*, *C*. *taina*, *Coe*. *productus*, *Hy*. *royesi*, *Meg*. *armaticeps* and *Meg*. *droegei.*

#### Distribution of the Effort of Collects Over a Century

**Fig. 6:**
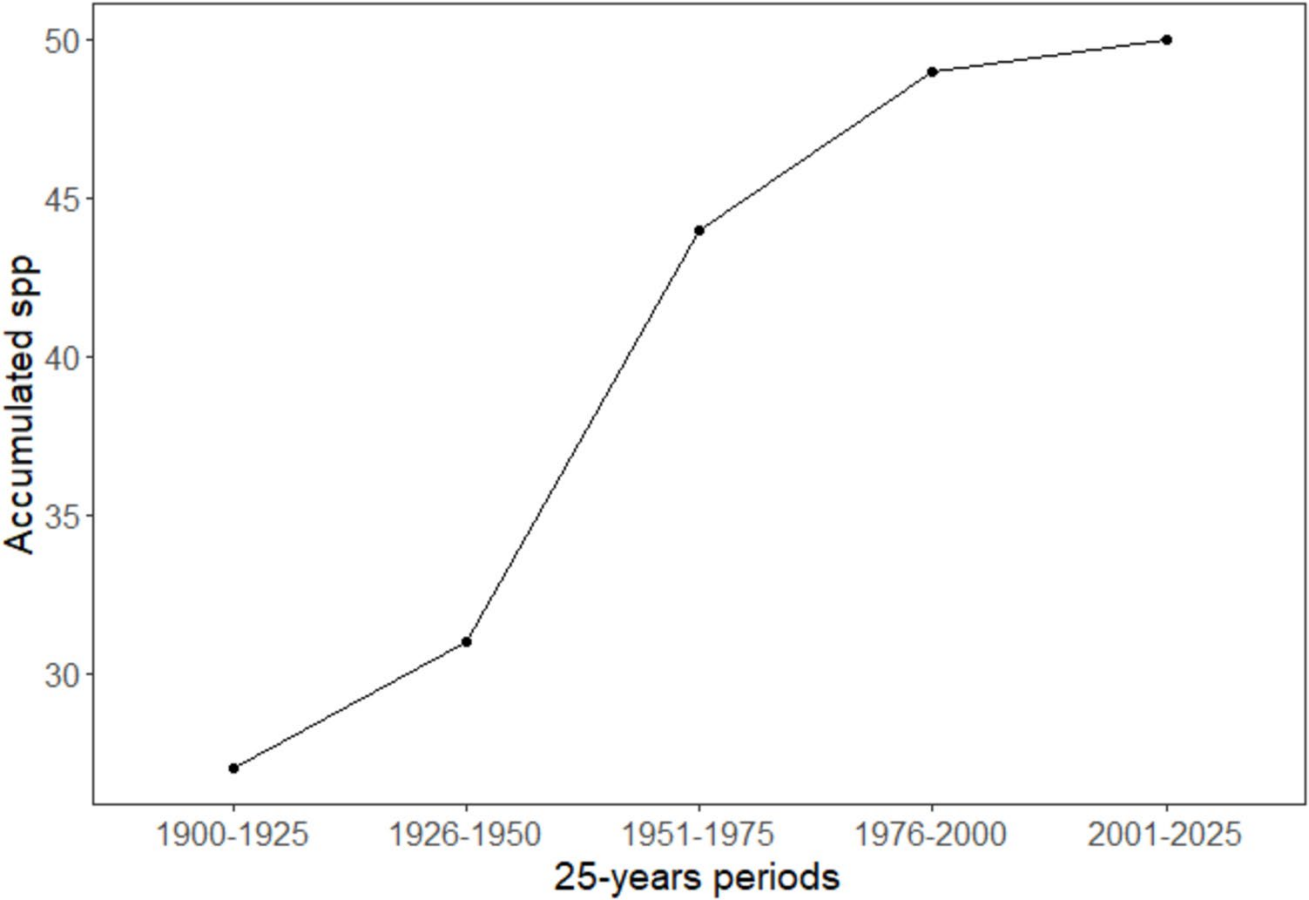
Species accumulation curve of wild bees through four successive 25-year periods between 1900 and 1999

Species sampling, and our knowledge of their richness, relative abondance and ecology, have increased significantly over the past century, but have not yet reached a plateau. In the initial 25-year period 1900–1925, 26 species of wild bees were documented in Havana, Mayabeque and Artemisa provinces (Fig. 6). This number increased steadily throughout the century to the current count of 52 species. Sampling in the period 1951–1975 was remarkably extensive compared to the preceding 25-year period. No significant differences were found between the periods in terms of species richness and abundance, with the exception of the 25-years period, before 1900, which showed significant differences with the rest of the group (Table 1).

**Table 1:** Temporal variation in species richness and individual abundance of wild bees in western Cuba

Time period	Species richness	Number of records
Before 1900	3	9
1900–1925	29	205
1926–1950	19	61
1951–1975	40	499
1976–2000	38	272
2001–2025	19	156

Over the years, the collecting effort fluctuated (except in the second period) and the visited sites varied from one period to another. This effort was lowest in the period 1926–1950 and the highest number of collecting events was between 1951–1975. The successive periods exhibited the following number of collect events: 205 during 1900–1925, 61 during 1926–1950, 499 during 1951–1975, 272 during 1976–2000, and 156 during 2001–2025.

**Fig. 7:**
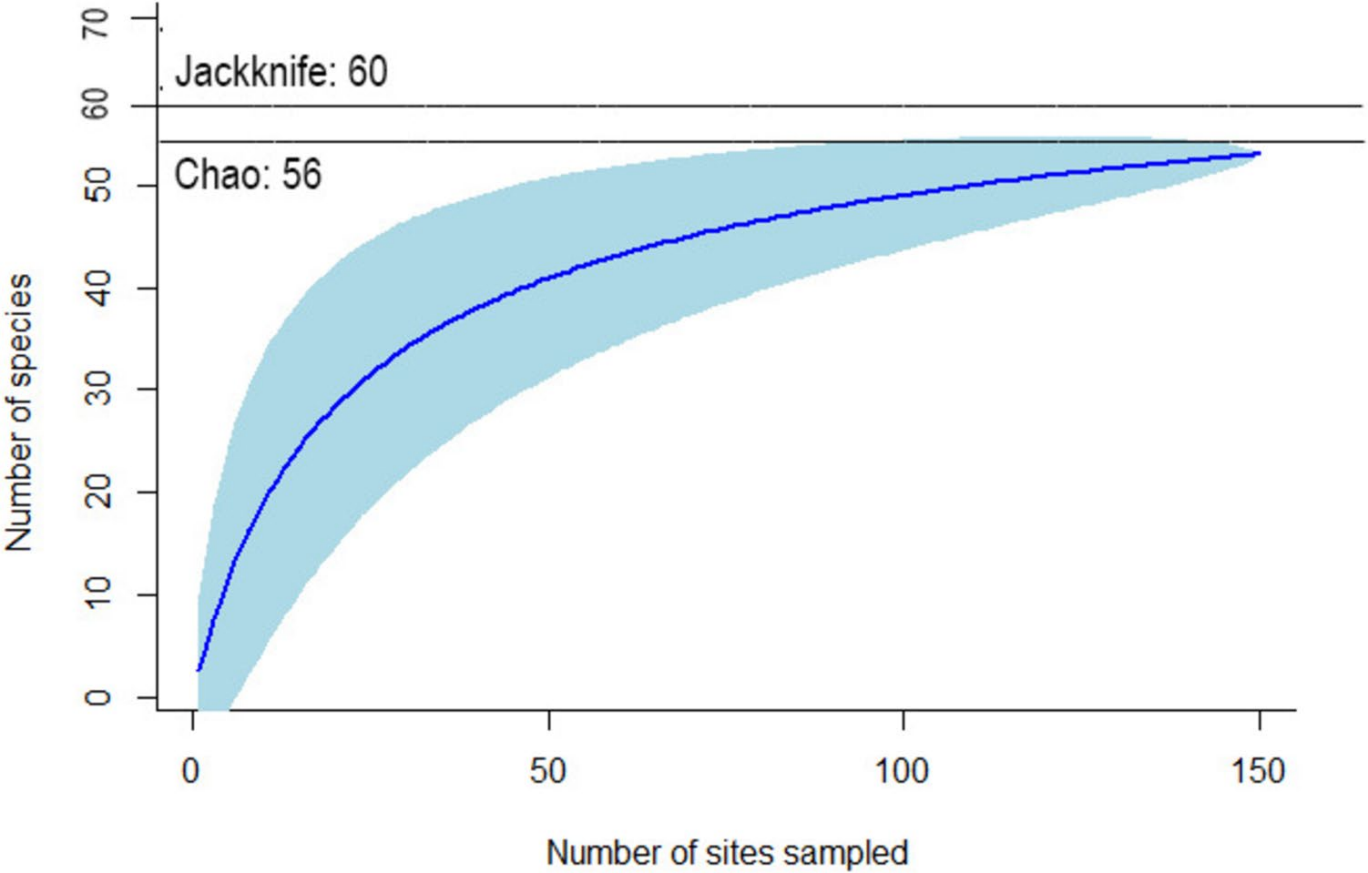
Species accumulation curve combining captures from all study years. The blue line represents the asymptotic model on the species accumulation curve, and the black lines represent the Jackknife and Chao estimation of richness

A species accumulation curve describing the richness of species through the century shows a cumulative increase in species richness as more years are sampled, with a final number of species of 52. The maximum expected number of species is estimated at 56 (± 4) with the Chao estimation (Fig. 7), and 60 (± 4) according to Jackknife. We recorded 92.9% and 86.7%, respectively, of the maximum predicted species count.

#### Feeding Habit of Species Over the Years

**Fig. 8:**
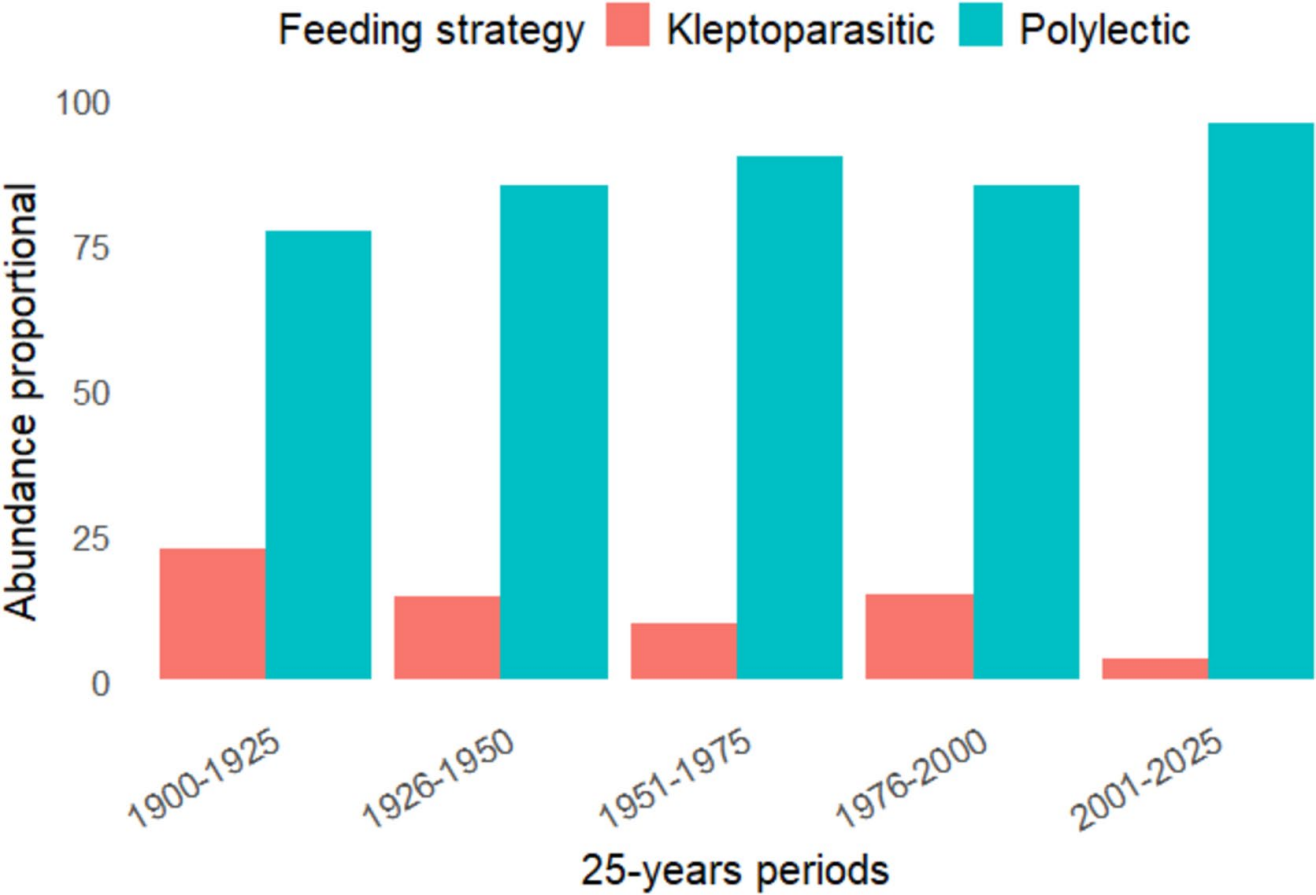
Proportional abundance of polylectic and kleptoparasitic wild bee species over five 25-year intervals in western Cuba

Data from the four 25-year periods indicated a systematically higher presence of polylectic non parasitic species compared to kleptoparasitic species (Fig. 8). During 1900–1925, kleptoparasitic species reached their highest proportional abundance (22.7%), relative to polylectic species collected in the same period. However, this value decreased progressively in the following two periods, decreasing by 8% to 14.6% in 1926–1950 and even more to 9.77% in 1951–1975. A slight recovery was observed in 1976–2000, with an increase in kleptoparasitic species close to 15%. However, the downward trend resumed in the last period (2001–2025), when kleptoparasitic species reached the lowest proportional abundance ever recorded (3.85%).

#### Seasonal Distribution of Species Over the Years

**Fig. 9:**
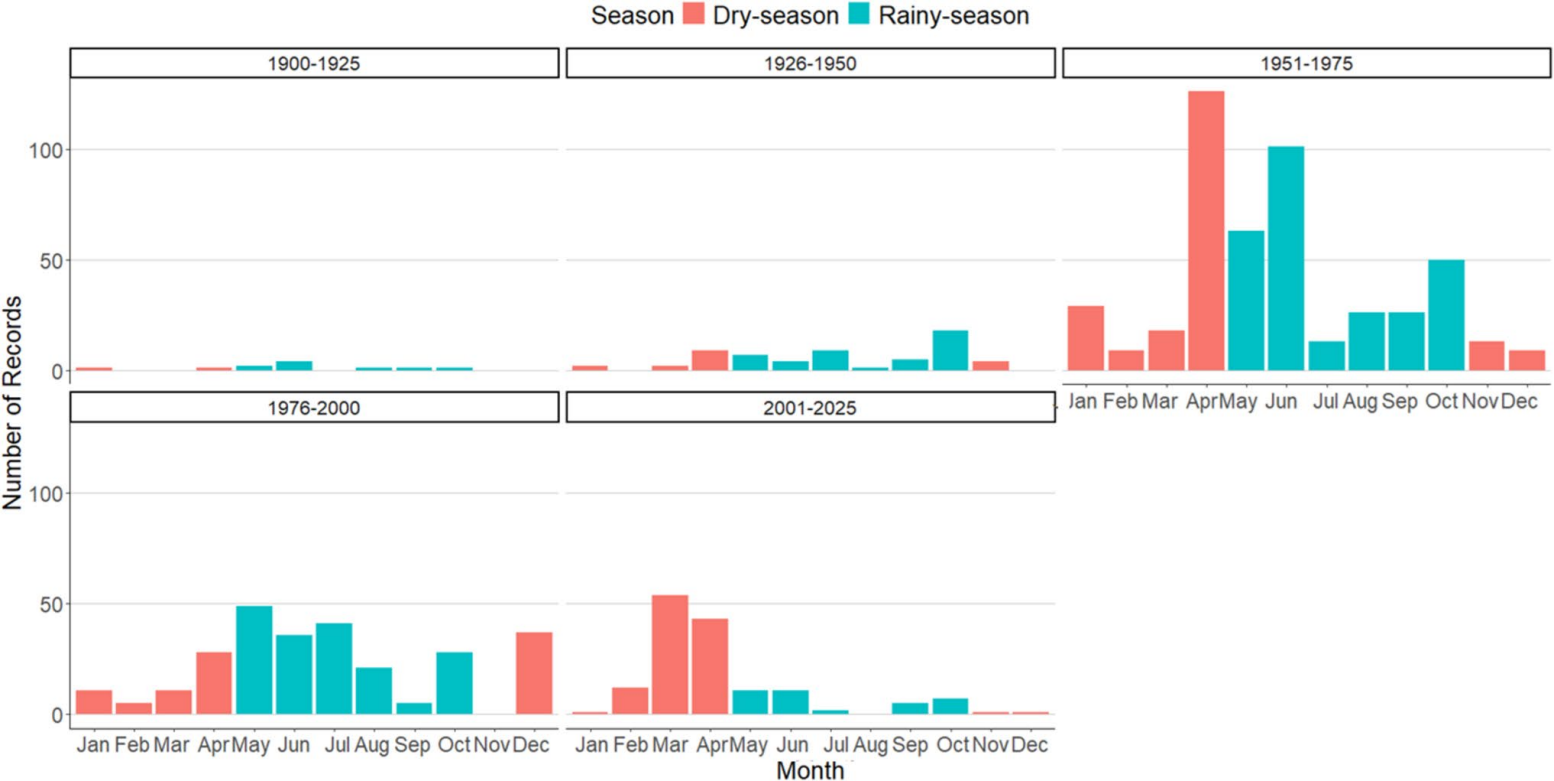
Seasonal distribution of the wild bee community throughout the surveyed time periods in the three western provinces of Cuba

The distribution of sampling across the four 25-year periods (Fig. 9) reveals notable trends and imbalances in collection efforts over time. During the earliest period (1900–1925), nearly all records (99%) were collected during the rainy season, with only minimal sampling in the dry season. A similar, though slightly less pronounced, pattern was observed in the second period (1926–1950), where 72.1% of the collections occurred during the rainy season. In contrast, sampling efforts in the following periods (1951–1975, 1976–2000, and 2001–2025) were more evenly distributed across seasons, reflecting a shift toward capturing data in both rainy and dry conditions. For instance, in 1951–1975, 44% of collections were taken during the dry season, and in 1976–2000, dry season collections reached 50%. All species were recorded in both seasons.

In terms of seasonal patterns, the rainy season consistently showed higher sampling effort, particularly in the months of May and June. For example, in the period 1951–1975, 101 records were collected in June, while May accounted for 63 records. During the same period, the dry season saw its peak in April, with 126 records collected. Similar trends persisted in the later periods, though at lower magnitudes. Between 2001–2025, March (dry season) showed a notable increase in collections, with 54 records, while April and June (rainy season) contributed 43 and 11 records, respectively.

## Discussion

### Diversity of Wild Bees

Our study revealed a rich community of bee species in the three provinces under investigation in the western region of Cuba, with 52 recorded species. The highest species richness (24 spp) was observed in the Apidae family, followed by Halictidae (15 spp), with the genus *Lasioglossum* being the most diverse within the latter (8 spp.).

These results correspond are congruent with the inventories conducted in the western (Genaro 2004; Breto 2021a, 2021b) and eastern parts of Cuba (Portuondo and Fernández-Triana 2004; Maceira et al. 2005). The genera such as *Apis*, *Ceratina*, *Melipona*, and *Xylocopa* are present in abundance within urban environments due to their functional traits: cavity nesting bees, generalist diet, and longer flight period spanning the two seasons (Genaro and Lóriga 2018; Buchholz and Egerer 2020).

In this research, five exotic species were detected: *Meg. concinna*, *Meg. lanata*, *Meg. rufipennis*, *A. mellifera* and *Mel. beecheii*. The last two are used for honey production in Cuba and cared for their survival (Genaro 2008; Genaro and Lóriga 2018). Alien species can directly or indirectly affect native ecosystems and thus become invasive (Russo 2016). The magnitude of the impact of an invasive bee species is related to its population size in the introduced habitat, competition for resource acquisition, and possibly, competition for nests as suggested by Zakardjian et al. (2022) in their case study with *Megachile* (*Callomegachile*) *sculpturalis* Smith in France. However, according to personal communications with the *Centro de Investigaciones Apícolas* (La Lisa, Havane), a unit specializing in science and technical expertise in Cuban apiculture, colonies of these species are located in sparsely urbanized areas, away from the city center.

Thirteen endemic species were recorded (two more species), and three species were identified as being in Critical (CR) according to the Red List of Cuba. These results are encouraging and testify to the fact that urban environments can harbor a non-negligible bee fauna (Baldock et al. 2015, 2019; Banaszak-Cibicka et al. 2018; Theodorou et al. 2020; Zaninotto and Dajoz 2022). Urbanization negatively affects the composition and diversity of bee communities (Potts et al. 2010; Bates et al. 2011; Fortel et al. 2014; Geslin et al. 2016; Hamblin et al. 2018). Yet in this study, where three provinces with high urbanization rates were sampled, we can still find 50% of the island's overall bee diversity.

### Origin and Distribution

As for plants (López Almirall 1998a, b, c), wild bees, especially endemic ones, are predominant in areas with more complex relief (Genaro 2008; Duarte and López 2019). Habana, Artemisa and Mayabeque provinces are characterized by a landscape with little complexity, except for La Habana, which presents a complex relief in the area of the Plain Habana-Matanza (Morrone and Crisci 1995; Duarte and López 2019). These results show that the Havana bee community is more diverse than those of Mayabeque and Artemisa.

An important limit in the explanations of our observations from historical collections is related to the fact that these data are largely opportunistic collect events, not performed in the frame of a well-defined protocol. It depended on the researchers who did the collect, and the selection of locations responded to different factors (proximity to their residences, vacation spots, collect in a specific area for some personal reason) (Genaro J. *pers. comm*. 2024). For example, most collections were made in the current province of La Habana, where the main collectors reside and/or work (Online resource1).

In La Habana, the area with the highest collect is Santiago de Las Vegas in Boyeros (157 records, 36 spp.). The high number of samples from this area could be related to the presence of large research centers (e.g., IES). IES has historical collections, and its researchers, who came from other academies (ECURED 2024), began collecting in the vicinity of the center from its inception. Additionally, these areas are less urbanized, featuring large and open natural spaces. In Mayabeque and Artemisa, the places with the highest collects correspond to sites considered as natural areas where urbanization was just beginning and access was quite easy, near Havana and areas of scientific interest for the Ministerio de Medio Ambiente in Cuba (Alayón J. *pers. comm*. 2024).

### Ecological Characteristics of the Wild Bees

The predominant feeding habits in the community of wild bees were primarily generalist (polylectic). This could imply the existence of selective filters that allow the presence of species with specific dietary preferences (Buchholz et al. 2020; Fauviau et al. 2022). The detection of kleptoparasitic species in the bee community is intriguing. Indeed, these species are known to play a stabilizing and indicative role in the community by being the first to react to disturbances (Sheffield et al. 2013a). The diversity and abundance of kleptoparasites relative to the total bee community may serve as indicators of the community state and its evaluation. The presence of kleptoparasitic bees also allows inferences about the presence of other species (on which they depend) in the community (Sheffield et al. 2013b).

Examining the flight phenology of pollinators is important as it allows for an understanding of the community and the development of protocols to assess and cover the entire pollination period in an area (Zaninotto and Dajoz 2022). Most species exhibited a long flight period, being present in the two seasons of the year. In Cuba, more than 400 plants that can provide resources to bees have been described, although it is suggested that only a few of them are harvested or most visited (Lóriga et al. 2020). Analyzing the phenological calendar of some of these flowering plants (Hechavarría et al. 2000; Castell Puchades et al. 2020; Lóriga et al.^2020^) highlight that the majority of the melliferous plants are most visited by bees between February and April, with March being the peak of abundance for flowering honey plants.

Taking into account this blooming peak, the highest number of wild bee species were recorded in the month of April (139), followed by May (102). Overall, wild bee abundance rise in the rainy season than during the dry season, influenced by climatic variables (Buchholz and Egerer 2020). During these periods, temperatures can vary from 15–20 °C in the dry season to 22–27 °C in the rainy season, indicating a warmer rainy period (Instituto de Meteorología de la República de Cuba (INSMET), 2024). Some wild bee species (genera *Ceratina*, *Lasioglossum*, *Xylocopa*, etc.) are favored by the higher temperatures of cities, which could explain the high abundance of these and other species in urban areas (see Hamblin et al. 2018).

However, as we didn’t observe significant trends between dry and rainy season, this could be also associated to the permanent resources offered to bees in the city with the cultivation of native and exotic flowering plants in private gardens as ornaments (Baldock 2020; Staab et al. 2020; Zaninotto et al. 2020). This is exemplified in the municipality of Regla, a highly urbanized area of Havana (ONEI 2022), where more than 80 flowering plants were found in domestic gardens, indicating that resources for pollinators are assured through this management approach (López-Almirall et al*.* in press).

### Persistence and Decline: A Temporal Perspective on Wild Bees

The patterns in wild bee diversity over 125 years reflect biases in historical collection methods, climate-driven environmental changes, and significant land-use transformations in Cuba. Historical collections, while valuable, are limited by geographic and taxonomic biases and the lack of structured protocols (Bartomeus et al. 2011). They often overrepresent dominant species and those in accessible habitats, leaving gaps for rarer taxa (Hughes et al*.* 2021). This may explain the frequent collection of species like *A*. *mellifera*, *Mel*. *beecheii*, *A*. *femoralis*, *H. poeyi*, and *C*. *cyaniventris* over others with specific ecological traits.

Since 2017–2018, a small group of naturalists has renewed insect collection and sharing through platforms like iNaturalist, enhancing knowledge of species distributions despite limited sampling (Jamieson et al. 2019). These efforts have identified declining species and documented new occurrences in specific regions. A study conducted in southeastern Michigan and northeastern Colorado by Jamieson et al. (2019) shows how in nine days of sampling, new occurrence records were documented at the county, region, and even state level.

Other factors influencing species distributions and declines over time include climatic variations and land-use changes (Bartomeus et al. 2011; Birdshire et al. 2020). Between 1971 and 2009, western Cuba experienced rising average maximum temperatures, with minimum temperatures exceeding 20 °C, and an increased frequency of precipitation events over 100 mm (Burgos Fonseca & González García 2012; Fernández-Alvarez et al. 2020). These changes have critical implications for local ecosystems, affecting bee activity and the flowering of the plants they pollinate (Burgos Fonseca & González García 2012).

Climate change affects bees differently depending on their traits: smaller, ground-nesting, and generalist species tend to increase in abundance with higher temperatures, while larger and cavity-nesting species often decline (Pardee Gabriella et al. 2022). In Austria, warmer and drier winters were associated with increased winter mortality in *Apis mellifera* colonies (Switanek et al. 2017). Similarly, heavy rainfall can affect different castes within a colony in varying ways (Cebotari & Buzu 2023). These climatic shifts are reshaping pollinator communities, with potentially significant consequences for ecosystems and agriculture (Switanek et al. 2017; Burgos Fonseca & González García 2012).

The response of bees to land use change varies according to the species and the magnitude of the change (Cariveau & Winfree 2015). According to Nuñez-Penichet et al. (2024), in Cuba land use for agriculture has decreased between 1985 and 2020, but urbanization has increased (ONEI, 2021), resulting in more artificial surfaces, which could reduce the presence of ground-nesting insect species (Bates et al. 2011). However, other studies have found that many bee species can maintain stable populations in urban areas (Fortel et al. 2014; Theodorou et al. 2016). This is partly due to green spaces, as man-made environments such as urban farms and gardens can support a rich and abundant flora (González Gutiérrez 2022; Puppo et al. 2023; López-Almirall et al*.* in press) and wild bee fauna (Matteson et al. 2008; Fortel et al. 2014; Egerer et al. 2019; Duarte et al. in preparation). This could explain the predominance of generalist, solitary and small-sized species such as *C. cyaniventris* and *H. poeyi*.

## Conclusions

This study summarizes the available information on the diversity of the wild bee community, its ecological characteristics, and its evolution over one century in the three western Cuban provinces since 1867. Ecological trait analyses identified a community with high richness of native and endemic species, and well-established groups, i.e., polylectic species more abundant in rainy seasons. We found evidence of sampling changes in species richness and abundance, with an increase in collections from the 1950 s onwards. This study has provided the first checklist of wild bee species in Havana, Mayabeque, and Artemisa. This will serve as a baseline for future studies on pollinator ecology in Cuba and the Caribbean region with de novo collecting events through a well-define protocol.

## Electronic supplementary material

Below is the link to the electronic supplementary material.

Supplementary file1 (XLSX 330 KB)

## Data Availability

The datasets generated and analyzed during the current study are available in the INDORES data repository at the following 10.48579/PRO/MLVPNI.

## Appendix 1

Table 2

**Table 2:** Number of individuals per institution and information on how data was collected at each museum. MNHNC: Museo Nacional de Historia Natural de Cuba, IES: Instituto de Ecología y Sistemática (La Habana, Cuba), MNHN: Muséum national d´Histoire naturelle (Paris, France), PCYU: Canada, Ontario, Toronto, York University, Packer (Canada), AMNH: American Museum of Natural History, SI-NMNH: Smithsonian Institution, National Museum of Natural History, Washington, KU-NHM: Kansas University, Natural History Museum, Kansas Collection, FSCA: Gainesville, Division of Plant Industry, Florida State Collection of Arthropods, Florida, LACM: Los Angeles, Los Angeles County Museum of Natural History, California, CUIC: Cornell University Insect Collection, New York, Ithaca, INHS: Illinois Natural History Survey, Illinois, Champaign, USNM: Washington D.C., National Museum of Natural History, Illinois, Champaign, MCZC: Harvard University, Museum of Comparative Zoology, Massachusetts, Cambridge, United State of America

COLLECTION	RECORDS	TAKEN FROM
MNHNC	611	Duarte (curator) 444 records and collecting between 2018–2019 and 2023–2024(165 records), literature (1 record)
IES	301	Send by Genaro
MNHN	224	Duarte (curator and labeling)
KUNHM	92	Museum website
iNaturalist	50	iNaturalist
USNM	13	GBIF
SI-NMNH	10	Museum website
AMNH	8	send by Smith and Rozen
MCZ	5	GBIF
INHS	2	GBIF
LACM	2	GBIF
CUIC	1	GBIF
FSCA	1	GBIF
NMNH	1	GBIF
PCYU	1	GBIF

## Appendix 2

Table 3

**Table 3:** List of the 52 wild bees present in La Habana, Artemisa, and Mayabeque, organized alphabetically by family, genus, and species, following the taxonomy of Michener (2007). Fh: Feeding habit: P (polylectic) and K (kleptoparasitic), St: status: N (native), * (endemic), and I (introduced), Distr.: Biogeographical distribution: Pan: Pangeographic, Nt: Neotropical region, Nr-Nt: Neotropical and Nearctic region, P1-4: the four 25-years periods (P1: 1900–1925, P2: 1926–1950, P3: 1951–1975, P4: 1976–2000, and P5: 2001–2025), S: season: 1: dry season, 2: rainy season, and 3: present in both seasons

SPECIES	Fh	St	Distr	P1	P2	P3	P4	P5	S
**Family Apidae**									
*Apis mellifera*	P	I	Pan		x	x	x	x	3
*Anthophora* (*Mistacanthophora*) *atrata*	P	N*	Nt	x			x		2
*Centris* (*Xanthemisia*) *aethiops*	P	N	Nt			x		x	2
*Centris* (*Centris*) *fasciata*	P	N	Nt			x	x		2
*Centris* (*Centris*) *poecila*	P	N	Nt			x	x	x	3
*Centris (Heterocentris) cornuta*	P	N	Nt	x					-
*Ceratina* (*Zadontomerus*) *cyaniventris*	P	N*	Nt		x	x	x	x	3
*Ceratina* (*Ceratinula*) *cockerelli*	P	N	Nr-Nt			x		x	2
*Epeolus pulchellus*	K	N*	Nt				x		3
*Exomalopsis* (*Exomalopsis*) *pulchella*	P	N	Nr-Nt			x		x	1
*Exomalopsis* (*Exomalopsis*) *similis*	P	N	Nr-Nt			x	x	x	3
*Florilegus* (*Florilegus*) *lanierii*	P	N	Nr-Nt	x	x	x	x	x	3
*Melipona* (*Melikerria*) *beecheii*	P	I	Nr-Nt			x	x	x	1
*Melissodes* (*Melissodes*) *leprieuri*	P	N*	Nt		x	x	x	x	3
*Melissodes* (*Melissodes*) *cubensis*	P	N*	Nt		x	x	x	x	3
*Melissodes* (*Eumelissodes*) *pullatus*	P	N*	Nt		x	x	x	x	3
*Mesoplia cubensis*	P	N*	Nt				x	x	3
*Nomada cubensis*	K	N*	Nt		x	x	x	x	3
*Nomada pilipes*	K	N	Nt			x	x	x	3
*Nomada cruralis*	K	N*	Nt	x	x	x	x		3
*Triepeolus vicinus*	K	N	Nt			x		x	2
*Triepeolus roni*	K	N	Nt				x		1
*Triepeolus wilsoni*	K	N	Nt	x			x	x	2
*Xeromelecta* (*Nesomelecta*) *alayoi*	K	N*	Nt				x		2
*Xylocopa* (*Neoxylocopa*) *cubaecola*	P	N	Nt			x	x	x	3
**Family Colletidae**									
*Colletes submarginatus*	P	N	Nt			x	x	x	3
*Hylaeus* (*Prosopis*) *limbifrons*	P	N*	Nt			x			3
**Family Halictidae**									
*Agapostemon femoralis*	P	N*	Nt			x	x	x	3
*Agapostemon* (*Agapostemon*) *poeyi*	P	N	Nt		x	x	x	x	3
*Auglochlora* (*Augochlora*) *elegans*	P	N	Nt		x	x			3
*Auglochlora* (*Augochlora*)* magnifica*	P	N	Nt						3
*Auglochlora* (*Augochlora*) *regina*	P	N	Nt	x		x		x	3
*Halictus poeyi*	P	N	Nt			x	x	x	3
*Lasioglossum* (*Dialictus*) *coreopsis**	P	N	Nr-Nt			x	x	x	3
*Lasioglossum* (*Dialictus*) *ferrerii*	P	N	Nt			x			3
*Lasioglossum* (*Dialictus*) *gundlachii*	P	N	Nt			x		x	3
*Lasioglossum* (*Dialictus*) *havanense*	P	N	Nt				x		2
*Lasioglossum* (*Dialictus*) *longifrons**	P	N	Nr-Nt					x	3
*Lasioglossum* (*Dialictus*) *mestrei*	P	N	Nt			x	x		3
*Lasioglossum* (*Dialictus*) *parvum*	P	N	Nt			x		x	3
*Lasioglossum* (*Dialictus*) *normale*	P	N*	Nt				x		3
*Nomia* (*Acunomia*) *robinsoni*	P	N	Nt			x	x	x	3
**Family Megachilidae**									
*Coelioxys* (*Neocolioxys*) *rufipes*	K	N	Nt			x	x	x	3
*Coelioxys* (*Cyrtocolioxys*) *tridentatus*	K	N	Nt	x					-
*Coelioxys* (*Boerocoelioxys*) *sannicolarensis*	K	N*	Nt				x		1
*Megachile* (*Sayapis*) *apora*	P	N	Nt				x	x	1
*Megachile* (*Eutricharaea*) *concinna*	P	I	Nr-Nt			x		x	2
*Megachile* (*Pseudomegachile*) *lanata*	P	I	Nr-Nt			x			2
*Megachile* (*Pseudocentron*) *poeyi*	P	N	Nt			x	x	x	3
*Megachile* (*Callomegachile*) *rufipennis*	P	I	Nt		x	x	x		3
*Megachile* (*Melanosaurus*) *singularis*	P	N	Nt			x	x	x	3
*Megachile* (*Leptorachis*)* curta*	P	N	Nt	x				x	3

^*^Should be re-examined, these two names were treated as synonyms for a long time
